# Cortical thickness across the cingulate gyrus in schizophrenia and its association to illness duration and memory performance

**DOI:** 10.1007/s00406-021-01369-2

**Published:** 2022-01-08

**Authors:** J.-W. Thielen, B. W. Müller, D.-I. Chang, A. Krug, S. Mehl, A. Rapp, H. Walter, G. Winterer, K. Vogeley, S. Klingberg, M. Wagner, T. Kircher

**Affiliations:** 1 Department of Psychiatry and Psychotherapy, LVR-Hospital Essen, Faculty of Medicine, University of Duisburg-Essen, Duisburg, Germany; 2 Department of Psychology, University of Wuppertal, Wuppertal, Germany; 3 Department of Psychiatry, LWL University Hospital Bochum, Ruhr University Bochum, Bochum, Germany; 4 Department of Psychiatry and Psychotherapy, University of Bonn, Bonn, Germany; 5 Department of Psychiatry and Psychotherapy and Center for Mind, Brain and Behavior (MCMBB), Philipps-University, Marburg, Germany; 6 Department of Psychiatry and Psychotherapy, University of Tübingen, Tübingen, Germany; 7 Division of Mind and Brain Research, Department of Psychiatry and Psychotherapy CCM, Charité-Universitätsmedizin Berlin, Corporate Member of Freie Universität Berlin, Humboldt-Universität Zu Berlin, and Berlin Institute of Health, Berlin, Germany; 8 Department of Psychiatry and Psychotherapy, University of Berlin, Berlin, Germany; 9 Department of Psychiatry and Psychotherapy, University of Cologne, Cologne, Germany; 10 Institute of Neuroscience and Medicine, Cognitive Neuroscience (INM-3), Research Center Jülich, Jülich, Germany

**Keywords:** Schizophrenia, Cingulate gyrus, Magnetic resonance imaging, Neuropsychological assessment

## Abstract

Schizophrenia has been associated with structural brain abnormalities and cognitive deficits that partly change during the course of illness. In the present study, cortical thickness in five subregions of the cingulate gyrus was assessed in 44 patients with schizophrenia-spectrum disorder and 47 control persons and related to illness duration and memory capacities. In the patients group, cortical thickness was increased in the posterior part of the cingulate gyrus and related to illness duration whereas cortical thickness was decreased in anterior parts unrelated to illness duration. In contrast, cortical thickness was related to episodic and working memory performance only in the anterior but not posterior parts of the cingulate gyrus. Our finding of a posterior cingulate increase may point to either increased parietal communication that is accompanied by augmented neural plasticity or to effects of altered neurodegenerative processes in schizophrenia.

## Introduction

Cognitive deficits are considered to be a primary characteristic of schizophrenia [69]. Retrospective studies showed cognitive deficits to be among one of the first prodromal signs in individuals who were later diagnosed with schizophrenia [32, 74]. Moreover, the importance of understanding cognitive dysfunction in schizophrenia is accentuated by the relative lack of cognition-specific treatment options [12]. Evidence suggests that there are discrete domains of cognitive impairment. An early study by Bilder et al. [8] reported mild to moderate deficits in verbal fluency, attention, processing speed and working memory but more severe deficits in executive functioning and episodic memory.

Studies assessing memory indicate that impairments in schizophrenia are common and disproportionately pronounced in comparison to the overall level of intellectual impairment [1, 23, 28, 61]. McKenna et al. [50] even proposed the existence of a “schizophrenic amnesia”. However, it has been suggested that some aspects of memory may be affected to a greater extent than others [1, 30]. For instance, studies in which tasks were matched for difficulty level, report greater impairment in recall as compared to recognition [1], verbal memory seemed to be more substantially impaired as compared to visual memory [73]. Whereas the different aspects of memory deficits in schizophrenia have been extensively studied, less is known about the course of memory dysfunction over time in schizophrenia. Initial evidence suggested that duration of illness, after removing variance related to e.g. age, is associated with several domains of memory [65, 69]. For instance, longer duration of the disorder was associated with worse performance on verbal [26, 69] and visual [26, 65], Cuesta et al. [17] episodic memory tasks as well as working memory tasks [22, 26]. To possibly localize these deficits on a neurobiological level, brain morphology has been studied extensively. In this regard, Hulshoff Pol and Kahn [35] reviewed longitudinal studies of brain anatomy in schizophrenia and reported progressive reductions in brain tissue over the first 20 years of the disorder, with schizophrenia subjects showing more than twice the rate of tissue loss as compared to controls.

Post-mortem studies in patients with schizophrenia provided evidence for neuroanatomical abnormalities, in particular the cingulate gyrus [7, 13, 72]. Moreover, in vivo neuroimaging studies comparing patients with schizophrenia to healthy controls have shown evidence of decreased gray matter volume in the posterior cingulate gyrus [36, 68], the anterior cingulate gyrus [31, 38, 66, 67], and across the entire cingulate gyrus [51, 53, 80]. Beckmann and colleagues [6] performed a connectivity based parcellation of the cingulate cortex and related the different sub regions to specific functions based on peak activations of 171 functional magnetic resonance studies. With respect to memory functions, they found two spatially distinct regions of increased neural activation, namely in the dorsal anterior and in the posterior part of the cingulate gyrus. Moreover, the authors showed that the anterior activation cluster overlay with activation seen in studies of error, reward and conflict monitoring while the posterior activation cluster overlay with regions associated with processing of spatial information and visual imagination. Given these findings, one may hypothesize that different aspects of memory decline in patients with schizophrenia are associated with gray matter abnormalities across different sub-regions of the cingulate gyrus that worsen with duration of the illness.

In the present study, changes in cortical thickness in the cingulate gyrus of patients with schizophrenia-spectrum disorder were investigated and compared to those of healthy controls. Given the aforementioned evidence that memory-related activation occurs throughout the anterior–posterior axis of the cingulate gyrus and that duration of illness is associated with memory performance, we assessed cortical thickness abnormalities in different sub-areas of the cingulate cortex of patients with schizophrenia-spectrum disorder and its relation to memory as measured with different neuropsychological tests, and the duration of illness.

## Methods

### Subjects

**Fig. 1:**
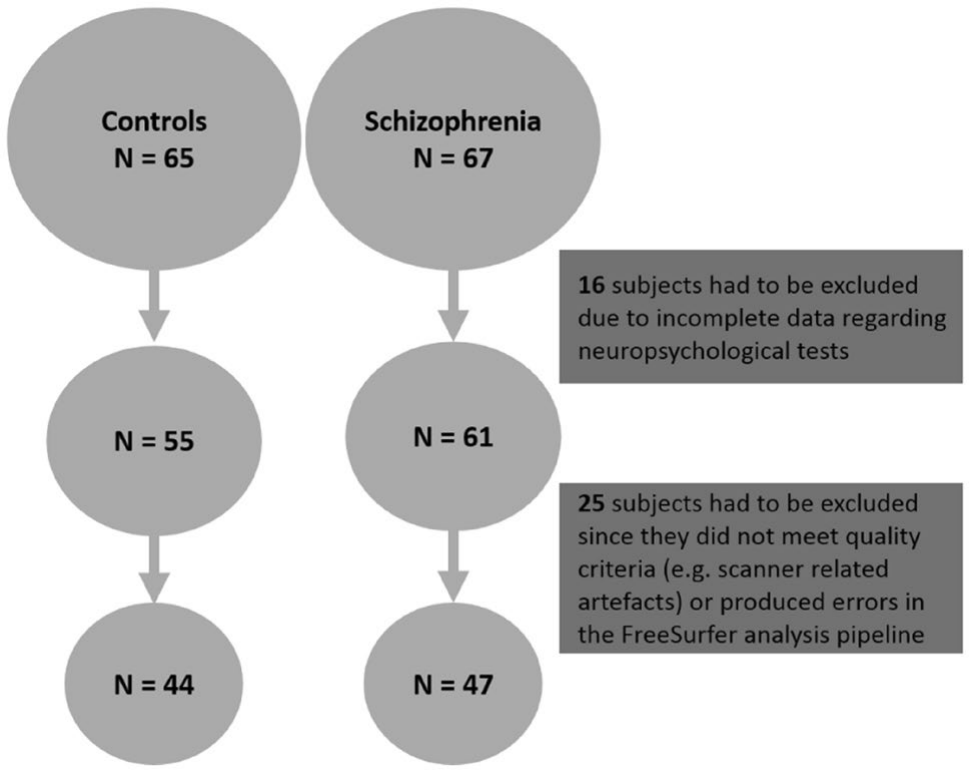
Flow chart

In the present study, we report data from the POSITIVE study, a randomized-controlled multicenter clinical trial on the effectiveness of a cognitive behavioral treatment for persistent positive symptoms (delusion and hallucination) in psychosis [40]. All study participants were asked to participate in an additional protocol on magnetic resonance imaging (MRI) [42, 43]. Data from 67 patients with schizophrenia-spectrum disorder and 65 healthy control subjects matched for age, sex and education were analyzed. Inclusion criteria for patients were a DSM-IV diagnosis of schizophrenia (DSM-IV 295.1, 295.2, 295.3, 295.6, 295.9), schizophreniform disorder (DSM IV, 295.4), schizoaffective disorder (DSM-IV 295.7), or delusional disorder (DSM IV 297.1), confirmed by a structured clinical interview (SCID-I), PANNS-P1 Delusion or PANNS-P3 Hallucination score ≥ 4 and persistent symptoms for at least 3 months. Patients and controls were recruited at six German university hospitals (Düsseldorf, Cologne, Bonn, Tübingen, Frankfurt and Essen). Controls had to have a life time history without psychiatric disorders, verified by a structured clinical interview (SCID-I, [81]. Overall, 41 subjects had to be excluded from the analysis because they had either incomplete data sets or produced errors in the FreeSurfer analysis pipeline or issues related to scan quality (details in Fig. 1). Quality assessment was done by visual inspection of the scans. Subjects were excluded if there were issues such as no full-brain coverage, artefacts as susceptibility, truncation, aliasing, chemical shift or ghosting and inadequate gray/white matter contrast.

A total of 47 patients with schizophrenia-spectrum disorder and 44 healthy controls were included and analyzed in the present study (see Table 1 for characteristics of the sample). All data reported here have been taken from the initial MRI assessment at the start of the psychotherapeutic intervention (see [40].

**Table 1:** Sample characteristics

	Schizophrenia	Healthy controls
	Mean (SD)	Mean (SD)
Gender (male/female)	37/10	28/16
Age (years)	37.49 (8.20)	35.57 (9.37)
Education (MWT-B)	27.17 (4.73)	31.33 (3.53)
PANNS (positive scale)	18.00 (4.18)	/
PANNS (negative scale)	15.09 (4.52)	/
PANNS (general scale)	32.74 (6.84)	/
Duration of illness (years)	12.50 (7.47)	/

*PANSS* positive and negative syndrome scale, *MWT-B* Mehrfachwahl- Wortschatz-Inteligenztest

### Clinical data

To account for potential differences regarding memory functions, we analyzed memory-related neuropsychological tests from the cognitive assessment battery in the POSITIVE study. These tests comprised the German Auditory Verbal Learning Test (VLMT; [33, 34, 48], the Digit Span Test [10], the Corsi Block-Tapping Test [16] and the Trail Making Test (TMT), Army Individual Test [3, 58]. These tests allow the assessment of short-term memory (holding information in mind), working memory (holding information in mind and manipulating it and long-term memory functions. The VLMT is the German equivalent of the Rey Auditory Verbal Learning Test (RAVLT; [59] and designed to assess verbal episodic immediate and delayed memory, the latter with free recall and cued recognition. The Digit Span Test covers verbal short-term memory and working memory [2, 5, 39, 46, 56, 63] and consists of a forward memory part and a backward memory part for number sequences. The Digit Span forward is assumed to be more related to short term memory whereas the Digit Span backward is more related to working memory [15]. The Corsi Block-Tapping Test [29] probes visual-spatial short-term and working memory. Blocks on a rectangular board are tapped, which the subjects have to immediately replicate forward or backward. The Corsi Block-Tapping Test is assumed to inform about spatial short-term and working memory, respectively. The TMT consists of two parts, TMT-A and TMT-B. In the TMT-A, subjects have to link numbers on a sheet of paper in ascending order. In part B numbers and letters have to be linked alternating in ascending order. TMT-A requires mainly visuoperceptual and motor speed abilities whereas the TMT-B reflects visuoperceptual, motor speed, working memory and executive functional abilities. Subtracting part A from B minimizes visuoperceptual and motor speed demands, providing a relatively better estimate of working memory abilities [47]. The Mehrfachwahl-Wortschatz-Test is a word identification test (MWT-B; [45]. Real words with increasing difficulty have to be identified among five alternatives. The MWT-B is utilized to estimate subject’s premorbid intelligence (verbal IQ).

To assess the relation of illness duration to potential alterations in cortical thickness and memory performance, we subtracted the subject’s age at the patient reported start of psychotic symptoms from the subject’s age at time of examination.

### MRI data acquisition

Image acquisition was performed on 3 T MR scanners (all Siemens Healthcare, Germany) at 4 sites, i.e. Tübingen, Frankfurt, Jülich and Düsseldorf (all Germany), assessing patients recruited at 6 university centers (Tübingen, Heidelberg, Frankfurt, Bonn, Düsseldorf and Essen). A T1 weighted MPRAGE sequence was used for structural image acquisition (sagittal) with a TR = 2.2 s, TE = 3.93 ms (minimal, asymmetric echo, bandwidth 130 Hz/Pixel), flip angle = 9°, slice thickness = 1.0 mm, slices per slab = 160, resolution (voxel size) = 1.0 mm^3^, FOV 256 mm × 256 mm.

### MRI data analyses

**Fig. 2:**
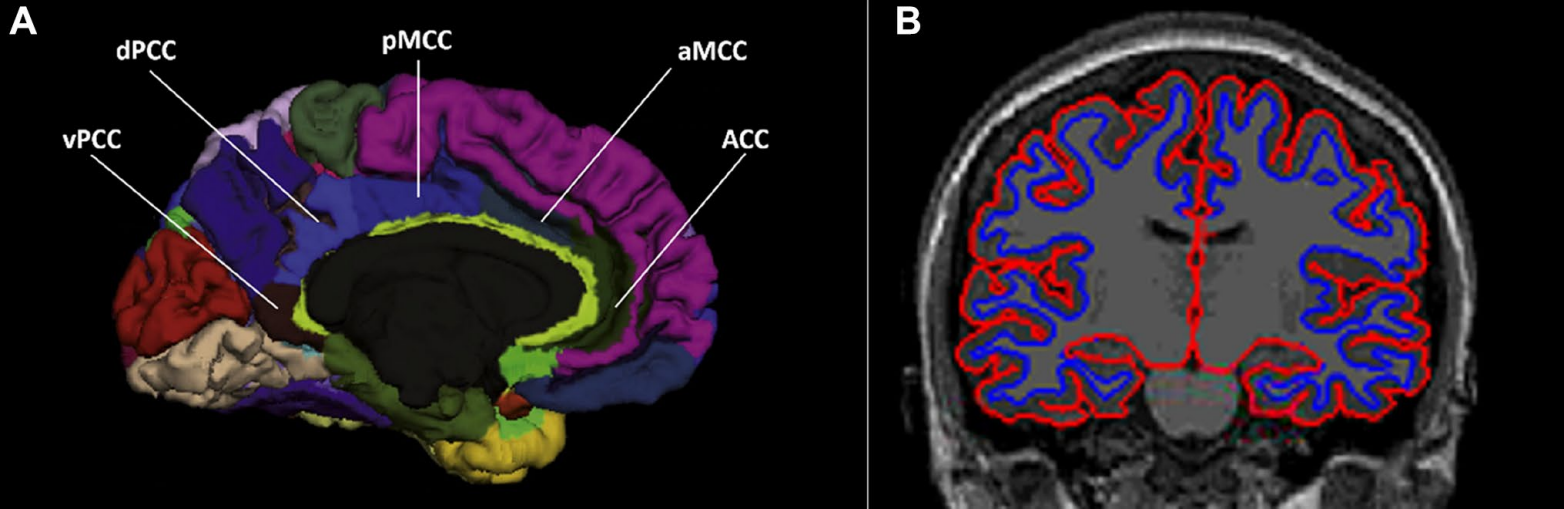
(**A**) The cingulate ROI’s are depicted. Anterior cingulate cortex (ACC; green), middle-anterior cingulate cortex (aMCC; gray), middle-posterior cingulate cortex (pMCC: dark blue), posterior-dorsal cingulate cortex (dPCC; light blue), posterior-ventral cingulate cortex (vPCC: brown). Figure adapted from [21]. (**B**) An image of cortical thickness is depicted. The red lines represent the pial surface and the blue lines represent the gray-white matter border. Cortical thickness is defined as the distance between the red and blue lines orthogonal to the pial surface

FreeSurfer image analysis suite (version 6.0) was used for structural data processing. (http://surfer.nmr.mgh.harvard.edu/). Cortical thickness measures, generated by FreeSurfer, have been validated both by histological [62] and manual measurements [44]. Technical details have been described previously in Fischl and Dale [25]. Briefly, cortical thickness measures were obtained by reconstructing representations of the pial surface and the white/gray matter boundary [19, 20] calculating the distance between those surfaces (Fig. 2B). Following automatic data processing, each image was manually inspected for errors in the processing pipeline.

### Description of regions of interest (ROI)

The 2009 (V6) version of the FreeSurfer package implements a new parcellation of the cingulate cortex (Destrieux Atlas/ aparc.a2009s atlas; [21] based on cytoarchitectonic and functional data. The cingulate sulcus, cingulate gyrus and intracingulate sulcus were grouped and subdivided in segments following the antero-posterior axis as proposed by Vogt and colleagues [77, 78]. This parcellation of the cingulate region includes an anterior (ACC), middle-anterior (aMCC), middle-posterior (pMCC), posterior-dorsal (dPCC), and posterior-ventral (vPCC or isthmus) subregion for each hemisphere (Fig. 2A).

### Statistical analysis

The cingulate specific (aparc.a2009s atlas) output from the FreeSurfer analysis was subjected to SPSS (IBM Inc.) software for statistical analysis. Multivariate analysis of covariance (MANCOVA) was conducted to identify differences in cortical thickness between the patients with schizophrenia-spectrum disorder and healthy controls. Gender, age, study center and mean cortical thickness served as covariates of no interest. Cortical thickness measures of the left and right ACC, aMCC, pMCC, dPCC, and vPCC served as independent and group as dependent variables. Associations of regional cortical thickness with duration of illness was assessed via Pearson’s partial correlation analyses controlled for age and mean cortical thickness. In a next step, memory performance and its relation to cortical thickness was assessed. A MANCOVA was conducted to reveal memory differences between patients with schizophrenia-spectrum disorder and healthy control persons. Here, the neuropsychological tests, VLMT (immediate recall, delayed recall, delayed recognition), Digit Span (forward, backward), Corsi Block-Tapping (forward, backward) and TMT (A, B, B-A), served as independent and group as dependent variables. To determine effects related to gender, age, study center and education (as measured with the MWT-B), we used these as covariates of no interest.

Pearson’s partial correlation between the neuropsychological tests and cortical thickness measures were calculated, controlling for age, mean cortical thickness and education. To reduce the amount of statistical tests, we assessed only associations between those cingulate subregions and neuropsychological tests that revealed a significant group difference in the initial MANCOVA analyses. In addition, we analyzed whether the neuropsychological tests that revealed a significant group difference were associated with illness duration. Therefore, we performed Pearson’s partial correlation analysis controlled for age and education. We used Bonferroni correction for multiple testing.

## Results

### MRI analysis

The omnibus test (MANCOVA) regarding cortical thickness differences across the cingulate sub-regions revealed a significant main effect of group between patients and healthy controls (*F* (10,76) = 1.931, *P* = 0.05). Post-hoc tests revealed significant differences between the independent groups: increased cortical thickness in the patients group in the right vPCC (*F* = 5.208, *P* = 0.025), and decreased cortical thickness in the patients group in the left ACC (*F* = 3.677, *P* = 0.05) and the left aMCC (*F* = 13.359, *P* = 0.001) (Table 2). However, if corrected for multiple comparisons (Bonferroni correction), only the difference in the left aMCC remains significant. While, the inclusion of covariates as age, gender and mean cortical thickness can dramatically influence results [37] we reanalyzed the data without these covariates and found that the effects in the left ACC remains stable (left ACC, *F* = 8.199, *P* = 0.005/left aMCC; *F* = 20.428, *P* < 0.001) but the effect in the right ACC turn to not significant (right vPCC; *F* = 3.231, *P* = 0.07).

**Table 2:** Mean cortical thickness, in patients with schizophrenia-spectrum disorder and controls

	Schizophrenia	Healthy controls	*F* value	*P* value
	Estimated marginal mean/SE (mm)	Estimated marginal mean/SE (mm)		
Right anterior cingulate (ACC)	2.555/0.019	2.548/0.020	0.051	0.822
Right middle-anterior cingulate (aMCC)	2.621/0.019	2.656/0.020	1.142	0.237
Right middle-posterior cingulate (pMCC)	2.556/0.017	2.598/0.018	2.575	0.112
Right posterior-dorsal cingulate (dPCC)	2.910/0.021	2.913/0.022	0.006	0.939
Right posterior-ventral cingulate (vPCC)	2.758/0.030	2.639/0.030	5.208	0.025*
Left anterior cingulate (ACC)	2.708/0.021	2.768/0.021	3.677	0.050*
Left middle-anterior cingulate (aMCC)	2.584/0.024	2.715/0.025	13.358	0.001**
Left middle-posterior cingulate (pMCC)	2.551/0.017	2.585/0.018	1.636	0.204
Left posterior-dorsal cingulate (dPCC)	2.983/0.023	2.987/0.023	2.054	0.155
Left posterior-ventral cingulate (vPCC)	2.700/0.041	2.660/0.042	0.423	0.517

Means represent cortical thickness in millimeters (mm). Compared to healthy controls, patients with schizophrenia-spectrum disorder showed reduced cortical thickness in the left anterior (ACC) and middle anterior cingulate (aMCC) and increased cortical thickness in the right posterior ventral cingulate (vPCC)

*Significant difference

**Significant difference (Bonferroni corrected)

### Analysis of neuropsychological tests

The omnibus test (MANCOVA) with neuropsychological tests as dependent variables revealed a significant main difference between patients and controls (*F* (10,76) = 4.884, *P* = 0.001). Post hoc tests showed significant differences in the adjusted means between the independent groups for the Corsi Block-Tapping backward, VLMT- immediate recall, VLMT- delayed recall, VLMT- recognition, TMT-A, TMT-B and TMT B-A. Compared to the healthy controls, patients performed worse on all these tests (Table 3). If corrected for multiple comparison (Bonferroni correction) all comparisons, except VLMT delayed recognition, remained significant.

**Table 3:** Estimated means of the neuropsychological tests, as corrected for gender, study center, age and education, of patients and healthy controls

	Schizophrenia	Healthy controls	*F* value	*P* value
	Estimated marginal mean/SE	Estimated marginal mean/SE		
Digit Span forward (amount correct)	7.767/0.317	7.784/0.330	0.001	0.973
Digit Span backward (amount correct)	6.920/0.346	7.714/0.360	2.199	0.142
Block-Tapping forward (amount correct)	8.157/0.310	8.343/0.322	0.150	0.699
Block-Tapping backward (amount correct)	7.362/0.297	9.009/0.309	12.843	0.001**
VLMT immediate recall (amount remembered)	9.465/0.437	11.991/0.454	13.989	0.001**
VLMT delayed recall (amount remembered)	8.378/0.463	12.084/0.481	26.826	0.001**
VLMT delayed recognition (amount remembered)	13.197/0.261	14.347/0.271	8.121	0.006*
TMT-A (seconds)	33.108/1.471	26.303/1.529	8.948	0.004**
TMT-B (seconds)	94.958/6.142	61.324/6.365	12.514	0.001**
TMT B-A (seconds)	61.850/5.582	35.020/5.803	9.660	0.003**

*Significant difference

**Significant difference (Bonferroni corrected)

### Correlation analysis

**Fig. 3:**
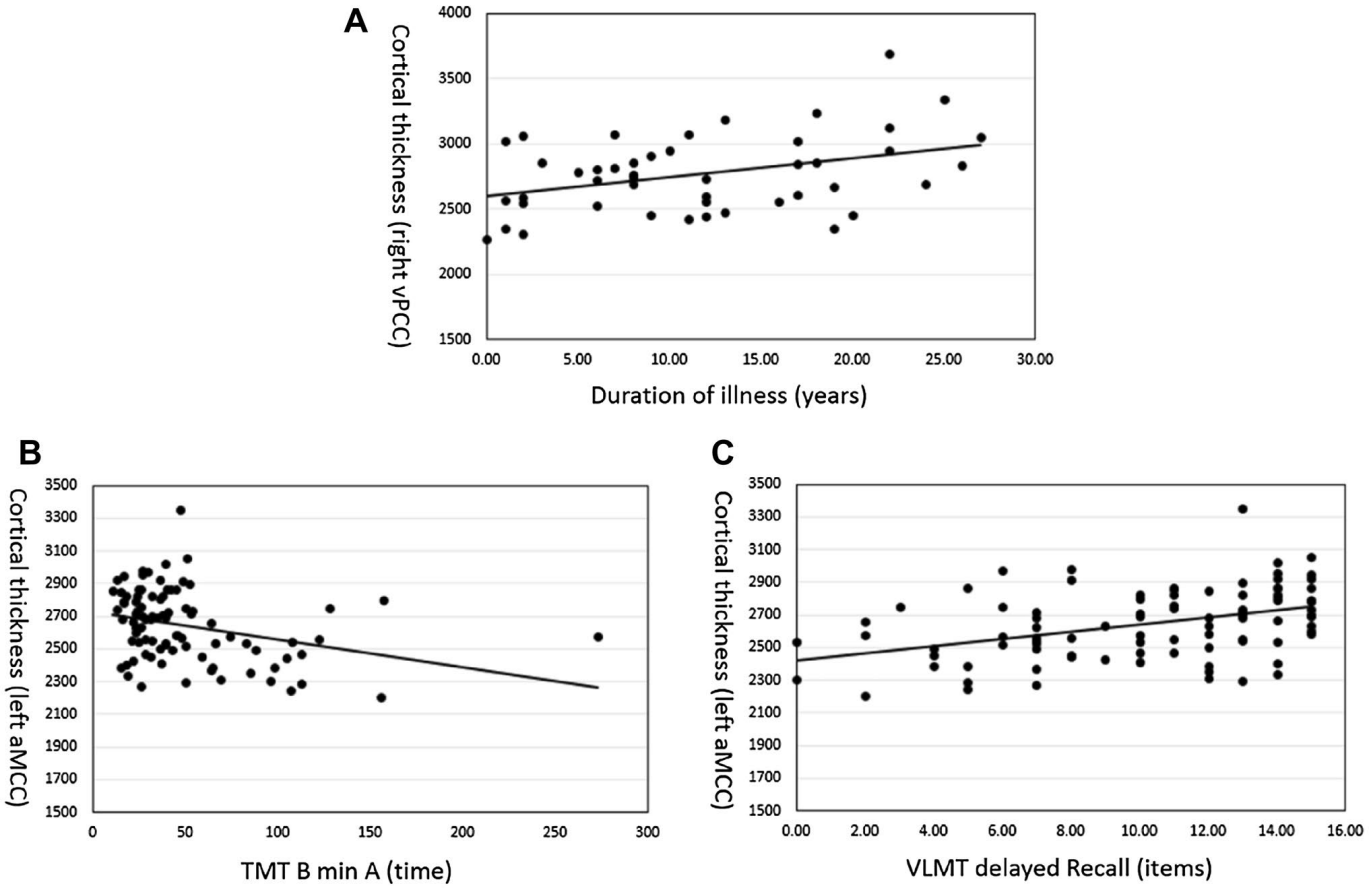
Scatterplots of the significant correlations. In (**A**) the correlation between right vPCC cortical thickness and duration of illness, in (**B**) the correlation between left aMCC and TMT-B min (**A**) and in (**C**) the correlation between left aMCC and VLMT delayed recall

Partial correlation analyses, revealed a positive association between duration of illness and cortical thickness measures in the right vPCC (*r* = 0.300; *P* = 0.050) but no associations with measures of left aMCC and ACC. The association between duration of illness and cortical thickness in the right vPCC remained even if additionally controlled for use of medication as measured with CPZ Equivalents. However, after correction for multiple testing (Bonferroni) no significant results remained. With regard to memory, partial correlation analyses in patients with schizophrenia-spectrum disorder revealed no correlation between duration of illness and the memory measures that appeared to be affected in patients. Regarding cortical thickness, we found a positive partial correlation between VLMT delayed recall performance and cortical thickness measures of the left aMCC (*r* = 0.232; *P* = 0.041). However, after correction for multiple testing (Bonferroni) no significant results remained. While the TMT (B minus A) data deviated from normal distribution, we performed Kendall's tau correlation and found negative correlation between TMT (TMT-B minus TMT-A) and cortical thickness measures of the left aMCC (*r* = − 0.317; *P* = 0.002; Fig. 3), which survived correction for multiple testing (Bonferroni). Similar to Assunção Leme et al. [4], there was no association between cortical thickness/performance measures and age of onset of the illness.

## Discussion

In the present study, we found that patients with schizophrenia-spectrum disorder exhibited both regional increase and decrease of the cingulate cortex in different sub-regions. In the posterior part of the cingulate gyrus, patients showed increased cortical thickness positively related to illness duration whereas the decreased cortical thickness in anterior parts of the cingulate gyrus appeared to be unrelated to illness duration. However, it should be noted that the finding of increased posterior cingulate cortical thickness did not survive the Bonferroni correction for multiple testing and should therefore be treated with caution. Nevertheless, this finding may point to a new hypothesis of an association between cortical thickness in the posterior cingulate and illness duration in patients with schizophrenia-spectrum disorder. Moreover, episodic and working memory deficits of patients with schizophrenia-spectrum disorder appeared to be associated with cortical thickness changes in the anterior but not posterior parts of the cingulate gyrus.

Consistent with previous studies, we found that patients with schizophrenia-spectrum disorder showed decreased cortical thickness in anterior parts of the cingulate gyrus. This is in accordance with findings from Van Haaren et al. [75] who reported decreased anterior cingulate thickness in patients with schizophrenia-spectrum disorder that continued to decrease over the time course of five years. Interestingly, similar to our findings of increased cortical thickness in the right vPCC, these authors showed that patients with schizophrenia-spectrum disorder exhibited increased cortical thickness in posterior parts of the brain such as parietal and occipital cortical areas. A hypothesis derived from these findings is that patients with schizophrenia-spectrum disorder may show a pronounced “communication” and neural processing within posterior brain networks, resulting in increased cortical thickness.

In line with this assumption is a recent finding by Wang et al. [80]. The authors studied resting-state-functional connectivity across several subregions of the cingulate gyrus in patients with schizophrenia-spectrum disorder. They reported that patients showed increased functional connectivity between posterior cingulate regions and parietal and occipital cortices, whereas anterior cingulate regions showed reduced functional connectivity to more frontal cortical regions. Functionally, Kronbichler et al. [41] reported decreased activation in anterior and increased activation in posterior brain regions of patients with schizophrenia during Theory of Mind tasks. In addition, empirical studies have demonstrated that activity of neurons can directly affect neurite outgrowth [76]. Taken together, the statistically uncorrected effect of increased cortical thickness in the vPCC may be taken to suggest that the “communication” of this region with others is increased, accompanied by augmented neuronal plasticity causing increased cortical thickness in posterior brain networks in which the vPCC functions as a hub. Another possible explanation may be related to maladaptive neurodevelopment in schizophrenia patients. The neurodevelopmental model of schizophrenia states that schizophrenia arises from insufficient synaptic pruning [24, 27, 57]. Insufficient synaptic or axonal pruning leaves more synapses or axon branches intact and could thereby cause cortical thickening in schizophrenia [82]. However, this seems unlikely given the positive correlation between vPCC cortical thickness and illness duration. Even though the exact pathological mechanisms of cortical thickening in schizophrenia needs further research, our finding of a statistically uncorrected vPCC thickening in patients with schizophrenia-spectrum disorder may provide a new hypothesis related to the pathology underlying schizophrenia.

Regarding memory, we evaluated memory deficits in patients with schizophrenia-spectrum disorder utilizing neuropsychological tests assessing working memory and different aspects of episodic memory such as immediate and delayed free recall and delayed recognition. Whereas working memory refers to the temporary storage of information and its manipulation and organization, recognition and recall refer to re-accessing of events or information from the past. Recognition refers to our ability to “recognize” an event or piece of information as being familiar, while recall designates the retrieval of related details of the memory. In line with previous studies (e.g. [52], we found that the patients with schizophrenia-spectrum disorder performed worse on neuropsychological tests of working memory (TMT-B minus TMT-A; CORSI block tapping backwards) and episodic memory (VLMT immediate and delayed recall). Regarding the association between cortical thickness and episodic memory performance we found a positive correlation between left aMCC cortical thickness and delayed recall of verbal material (VLMT-delayed recall). Note however, this effect should be interpreted with caution since it did not survive correction for multiple testing. Moreover, there was no association between cortical thickness and immediate recall (VLMT immediate recall). A possible explanation for the absence of an association between immediate recall performance and anterior cingulate thickness may be the remoteness of the memory. In this regard, functional studies in rodents and humans demonstrated that the anterior cingulate cortex is more involved in the retrieval of remote but not of recent memories [11, 70, 71]. However, with respect to cortical thickness, previous studies report inconsistent findings regarding an association between anterior cingulate cortical thickness and VLMT/RAVLT delayed recall performance. For instance, Chang et al. [14] reported no association between anterior cingulate cortical thickness and RAVLT delayed recall performance in patients with mild cognitive impairment whereas Gefen et al. [18] found a positive association in healthy elderly. These inconsistencies may at least partly be related to different parcelations of the cingulate cortex across these studies. Regarding vPCC, we could not find any associations between cortical thickness and episodic memory performance. A possible explanation may be that the neuropsychological tests used are not sensitive enough since functional MRI studies indicate posterior cingulate activation when participants performed episodic memory tasks [55, 60].

Regarding working memory, we found a negative correlation between TMT (TMT-B minus TMT-A) performance and left aMCC cortical thickness. This is similar to the finding by Gefen et al. [18], who reported a negative association between TMT-B raw sores and mean (left and right) anterior cingulate cortical thickness in the elderly. With respect to posterior cingulate cortical thickness we could not find an association with the different working memory tasks (TMT B minus A; Corsi block tapping backwards). However, functional imaging studies revealed evidence that both anterior and posterior cingulate regions are involved when participants perform the TMT task. For instance, Zakzanis et al. [82] showed increased activation in anterior and posterior parts of the cingulate gyrus in the TMT-B minus TMT-A condition in healthy young adults. Moreover, by using voxel-based morphology (VBM), Biundo and colleagues [9] revealed a negative association between TMT-B minus TMT-A performance and gray matter volume in both posterior and anterior cingulate gyrus. Moreover, MacPherson et al. [49] showed an association between cortical thickness in the isthmus of the cingulate cortex corresponding to vPCC and TMT-B performance in the elderly. However, MacPherson and colleagues included 411 healthy elderly and found a relation between TMT-B and a cluster located in the isthmus not the mean cortical thickness of whole isthmus as studied in the present study. Thus, sample size and/or the methodological analysis differences may explain these inconsistencies. In conclusion, we replicated that patients with schizophrenia-spectrum disorder exhibit memory deficits across different domains (delayed recall and working memory) and that these deficits are related to abnormal cortical thickness in the cingulate gyrus.

With respect to duration of illness, we could not find an association between duration and memory performance. Moreover, we found no association between duration of illness and anterior cingulate regions. However, as mentioned above, we found hints towards a probable association between duration of illness and right vPCC cortical thickness. This finding is not in line with the finding of Wang et al. [79]. Wang et al. reported a trend towards a negative association between duration of illness and both anterior and posterior cingulate cortical thickness. Moreover, Kuperberg et al. [44] reported a significant negative correlation between anterior cingulate cortical thickness and duration of illness whereas Nesvag and colleagues [54], Schultz et al. [64] and Assunção Leme et al. [4] could not find an association between cingulate thickness and illness duration. Thus, studies assessing the association between duration of illness and cingulate cortical thickness are controversial. This may be related to methodological differences in estimating cortical thickness, the parcellation of the cingulate sub-regions and different statistical procedures controlling for age, sex and education. Nevertheless, our finding of a (statistically uncorrected) positive relation between duration of illness and cortical thickness in the vPCC may indicate that there are specific neuronal processes that are changed during the time course of schizophrenia causing abnormal increased neuronal plasticity as opposed to neurodegeneration.

Some limitations should be acknowledged in this study. First, even though results were controlled for scanning site, this does not control for signal to noise ratio fluctuation between scanners over time. Therefore, noise due to changes in scanner profiles may have influenced the results [42, 43]. Second, patients were chosen based on their current psychopathology (predominant positive symptoms) and their interest in participation in a study on cognitive therapy in schizophrenia. While patients had a notable duration of illness, the validity of the results of our study may be confined to patients with some years in treatment and therefore not present in first episode patients. Third, we had to exclude some participants due to methodological issues. Fourth, this work compares many different variables leading to the issue of the possibility of type 1 errors. To prevent these, Bonferroni correction was applied which reduced significance of the results dramatically. Therefore, the validity of results that did not survive Bonferroni correction should be treated with caution. A strength of our study is the sampling of data within the context of a large phase III equivalent randomized controlled trial, with clinical ratings by dedicated researchers and professional data handling procedures [40].

Altogether we replicated findings of reduced cortical thickness in anterior parts of cingulate gyrus in patients with schizophrenia-spectrum disorder that are associated with both episodic and working memory deficits in these patients. Moreover, to the best of our knowledge, we showed for the first time that patients with schizophrenia-spectrum disorder may exhibit increased cortical thickness in the right vPCC that seems to be associated with duration of illness. Future studies may utilize functional MRI to assess whether patients with schizophrenia have pronounced neural processing within posterior brain networks and if this is related to cortical thickness of the isthmus cingulate.
